# Editorial: The Neonatal Immune System: A Unique Host-Microbial Interface

**DOI:** 10.3389/fped.2017.00274

**Published:** 2017-12-21

**Authors:** Joseph M. Bliss, James L. Wynn

**Affiliations:** ^1^Women & Infants Hospital of Rhode Island, Alpert Medical School of Brown University, Providence, RI, United States; ^2^University of Florida, Gainesville, FL, United States

**Keywords:** neonate immune responses, neonatal infection, sepsis, host–pathogen interactions, microbiome and immune system

**Editorial on the Research Topic**

**The Neonatal Immune System: A Unique Host-Microbial Interface**

The human infant is delivered at a stage in which every organ system is very much in the process of rapid development. As such, this stage of life is unique relative to any other that the organism will subsequently experience. This Research Topic focuses on the immune system of the newborn as one such organ system. The articles herein are original research or review articles that discuss some aspect of the neonatal immune system and/or the pathogens against which the system has evolved to defend. The articles can be broadly classified into three overall themes. (1) The influence of early life microbial and nutritional exposures on the formation and function of the human microbiome and mucosal immunity. (2) Characteristics of specific immune cell subsets that differ in the neonatal period relative to other life stages. (3) Specific neonatal infections, neonatal host-defense mechanisms, and unique features of the inflammatory response including vaccines. (4) Fungal infections and host defense in the newborn.

## Section 1: The Microbiome, Feeding, and Mucosal Immunity

The complexity and importance of the microbiome on human development, health, and disease has only begun to be appreciated. In this section, the microbiome and the influence of feeding early in life are reviewed with an emphasis on its influence in developing mucosal immunity.

### Early Life Host–Microbiome Interphase: The Key Frontier For Immune Development

Amenyogbe et al. summarize the interaction of the immune system with the microbiome in early life as a critical window of susceptibility for life long disease, as well as an opportunity to protect and promote life-long health.

### Starter Feeding Supplementation Alters Colonic Mucosal Bacterial Communities And Modulates Mucosal Immune Homeostasis In Newborn Lambs

An original study performed by Liu et al. shows that starter feeding altered colonic mucosal bacterial composition and modulated mucosal immune homeostasis, including TLR4 transcript as well as TNF-α and IFN-γ, during the milk-feeding period in lambs.

### Innate Immunity And Breast Milk

The ability of human milk to provide optimal growth and development to the newborn in ways that formula cannot has become increasingly apparent. In this review, Cacho and Lawrence focus on bioactive components of human milk that contribute to the developing innate immune system through unique and fascinating mechanisms including shaping the intestinal microbiome, diminishing inflammation, and providing pluripotent human milk stem cells that may have a role in tissue regeneration in the breastfed infant.

### The Role Of Mucosal Immunity In The Pathogenesis Of Necrotizing Enterocolitis

In this review, Hodzic et al. describe the cellular components of the intestinal epithelium and mucosal immune system as well as their relationship to NEC. They also discuss the relationship between the gut microbiota and cell signaling that underpins disease pathogenesis and highlight notable therapeutic advancements in NEC that target intestinal mucosal immunity.

## Section 2: Neonatal Innate and Adaptive Immunity

Although historically considered immature, deficient, or dysfunctional, more recent study of individual cellular components of the innate and adaptive immune system suggest that the alterations in function that are characteristic of neonatal cells reflect an evolutionarily adaptive phenotype rather than an immature one. Additionally, interactions between the cellular players have blurred the traditional distinction between the innate and adaptive arms. This section combines reviews and some original research into these phenomena.

### Age-Appropriate Functions And Dysfunctions Of The Neonatal Neutrophil

Lawrence et al. review that neutrophils are no longer viewed as short-lived, indiscriminate phagocytes of the immune system, but instead as essential components necessary for proper B and T cell function, antigen presentation, and tissue repair and regeneration. Unlike adult neutrophils, neonatal neutrophils are not simply “dysfunctional,” but rather exhibit phenotypic and functional variances required to tolerate the hypoxic *in utero* condition without triggering inflammatory responses, yet, remain able to mount sufficient pro-inflammatory reactions if exposed to pathogenic organisms.

### Rapid CD8+ Function Is Critical For Protection Of Neonatal Mice From An Extracellular Bacterial Enteropathogen

New research from Siefker and Adkins demonstrates that neonatal CD8^+^ cells, but not CD4^+^ cells, increase rapidly in proportion and IFN-γ production to primary infection with *Y. enterocolitica*, and play an important early, innate-like role in survival. However, using knockout mice, they show CD8^+^ cells were not necessary for the development of protective memory in neonates since they were dispensable for survival of secondary infection.

### Comparison of The Functional Microrna Expression In Immune Cell Subsets of Neonates and Adults

Yu et al. present novel systematic and functional miRNA profiles of neonate cord blood and adult leukocyte subsets. These studies serve as a basis to further understand the altered immune response observed in neonates and advance the development of therapeutic strategies.

## Section 3: Neonatal Sepsis, Meningitis and Vaccines

Neonatal sepsis and meningitis continue to carry a high burden of mortality and morbidity among those affected. Although attention to infection prevention practices has been effective at reducing risk of acquiring some of these infections, our approaches to treatment: administration of antimicrobial drugs, and supportive care, have remained essentially the same for decades. This section reviews new information about this problem garnered from modern techniques and presents some original research related to vaccine development and responses in neonates. Finally, the unique susceptibility of neonates to fungal infection is reviewed.

### Immunological Defects In Neonatal Sepsis And Potential Therapeutic Approaches

Raymond et al. review key distinctions of neonatal innate and adaptive immunity as compared to adults, summarize what has been learned using transcriptomic approaches on blood to uncover unique neonatal sepsis pathophysiology, and discuss why novel approaches unique to the neonate will be required for the development of both diagnostics and therapeutics in this population.

### Neonatal Meningitis: Overcoming Challenges In Diagnosis, Prognosis, and Treatment With Omics

In this contemporary review of a vexing problem, Gordon et al. discuss how modern “omics” technologies have been applied to study and further elucidate the complex interplay between infecting bacterial pathogens and the central nervous system in the setting of neonatal meningitis. Data generated from these studies are likely to be key components to a better understanding of pathogenesis, which is needed to improve diagnostic and therapeutic strategies and ultimately outcomes of this devastating disease.

### A Meningococcal Outer Membrane Vesicle Vaccine Incorporating Genetically Attenuated Endotoxin Dissociates Inflammation From Immunogenicity

In this original research article, Dowling et al. describe the unique and favorable profile of an outer membrane vesicle-based vaccine for *Neisseria meningitidis* derived from a mutant strain with attenuated endotoxin. This vaccine generated a considerably less inflammatory profile from human neonatal and adult leukocytes in whole blood assays *in vitro* compared to several currently licensed vaccines while still inducing a robust antibody response *in vivo*. These data suggest that *in vitro* systems may be useful to reflect age-specific inflammatory and immunogenic profiles of vaccine candidates as well as the properties of adjuvants in vaccine design.

### Immunization Of Newborn Mice Accelerates The Architectural Maturation of Lymph Nodes, But Aid-Dependent IgG Responses Are Still Delayed Compared to The Adult

This original research article by Munguía-Fuentes et al. describes the anatomic, cellular, and functional maturation that occurs in neonatal mouse lymph nodes following immunization on the day of birth. They describe recruitment of B cells and T cells, but less robust formation of germinal centers and reduced isotype switching compared to adults. The findings further define unique aspects of the murine antigen response in the early postnatal period that may be more accurately described as adapted to early life rather than immature.

## Section 4: Neonatal Fungal Infections

### Antifungal Immunological Defenses In Newborns

Michalski et al. present a review of the developing innate and adaptive immune responses of newborns, specifically as they may relate to susceptibility to fungal infections. Although much has been learned about the developing immune system in neonates and its relation to risk for infections, much less has been devoted to fungi as targets of host-defense. While highlighting antifungal host defense, the authors also describe important limitations of current studies.

### Investigation of Och1 In The Virulence of *Candida parapsilosis* Using A New Neonatal Mouse Model

Csonka et al. describe a method to investigate *C. parapsilosis* virulence in a newborn mouse model using intravenous infection *via* the facial vein in 2-day old mouse pups. This unique approach provides a novel contribution to available murine models of neonatal candidiasis. The similarity of this method to the well-established tail-vein method in adult mice allows more direct comparisons between the adult and neonatal response to bloodstream infection with *Candida* than previously described neonatal methods.

### *Candida parapsilosis* Protects Premature Intestinal Epithelial Cells From Invasion And Damage By *Candida albicans*

Gonia et al. investigate the potential for *C. parapsilosis* to modulate pathogenic interactions of *C. albicans* with the premature intestine. In this original work, they demonstrate that *C. parapsilosis* is able to reduce invasion, damage, and virulence functions of *C. albicans via* secreted molecules as well as by physical contact with the *C. parapsilosis* cell surface. This discovery brings the possibility that virulence features of pathogens can be modulated by other closely related species in the microenvironment.

## Author Contributions

Both authors participated in the editorial process for the entire Research Topic and wrote the accompanying editorial.

## Conflict of Interest Statement

The authors declare that the research was conducted in the absence of any commercial or financial relationships that could be construed as a potential conflict of interest.

